# Combined Circulating microRNA and Inflammatory Cytokine Profiles Improve Disease-Stage Discrimination of Charcot Foot in Egyptian Patients with Type 2 Diabetes Mellitus

**DOI:** 10.3390/biomedicines14040750

**Published:** 2026-03-25

**Authors:** Heba Ibrahim Hamed, Ihab Nabil Amin, Salwa Bakr Hassan, Ashraf Ismail Amin, Ibrahim Ali Emara, Heba Ramadan Ahmed, Lamis Safwat Mubarak, Shaimaa M. Abd El Aziz, Ahmed Abd Elrahman Elatreby, Ahmed Mohamed El Sabawy, Abeer Attia Saad, Mahmoud Gamal Algammal, Ahmed M. A. Akabawy

**Affiliations:** 1 Department of Biochemistry and Molecular Biology, Research and Training Unit, National Institute of Diabetes and Endocrinology, General Organization for Teaching Hospitals and Institutes, Cairo 11441, Egypt; prof.ibrahim.emara@gothi.gov.eg; 2 Department of Vascular Surgery, National Institute of Diabetes and Endocrinology, General Organization for Teaching Hospitals and Institutes, Cairo 11441, Egypt; doctorihabhanna@gmail.com; 3 Department of Clinical Pathology/Hematology and Transfusion Medicine, Faculty of Medicine, Fayoum University, Fayoum 63514, Egypt; 4 Department of Clinical Pathology, National Institute of Diabetes and Endocrinology, General Organization for Teaching Hospitals and Institutes, Cairo 11441, Egypt; ashraf.ismail@gothi.gov.eg; 5 Department of Internal Medicine, National Institute of Diabetes and Endocrinology, General Organization for Teaching Hospitals and Institutes, Cairo 11441, Egypt; hebar235@gmail.com; 6 Department of Rheumatology and Rehabilitation, Ahmed Maher Teaching Hospital, General Organization for Teaching Hospitals and Institutes, Cairo 11562, Egypt; lamissafwat25@gmail.com (L.S.M.); drshimaa.rehab@gmail.com (S.M.A.E.A.); 7 Department of Rheumatology and Rehabilitation, National Institute of Neuromotor System, General Organization for Teaching Hospitals and Institutes, Cairo 12411, Egypt; atr.1000@hotmail.com; 8 Department of Endocrinology, National Institute of Diabetes and Endocrinology, General Organization for Teaching Hospitals and Institutes, Cairo 11441, Egypt; aelsabawy@hotmail.com; 9 Department of Clinical Pathology, Faculty of Medicine, Ain Shams University, Cairo 11566, Egypt; abeerattia@med.asu.edu.eg; 10 Department of Internal Medicine, Faculty of Medicine, Fayoum University, Fayoum 63514, Egypt; mga11@fayoum.edu.eg; 11 Department of Biochemistry and Molecular Biology, Faculty of Pharmacy, Capital University (Formerly Helwan University), Ain Helwan, Cairo 11795, Egypt; dr.ahmed_moh@pharm.capu.edu.eg

**Keywords:** diabetic peripheral neuropathy, Charcot foot, microRNAs, inflammatory cytokines, type 2 diabetes mellitus, biomarker panel

## Abstract

**Background/Objectives:** Diabetic peripheral neuropathy (DPN) and Charcot foot (CF) represent progressive and disabling neuropathic complications of type 2 diabetes mellitus (T2DM). Circulating microRNAs and inflammatory cytokines may reflect underlying molecular alterations associated with disease progression and offer potential value for discriminating between stages of diabetic neuropathic complications. This study aimed to evaluate circulating miRNA expression profiles and inflammatory cytokine biomarkers in T2DM patients with and without neuropathic complications and to assess their potential non-invasive utility as combined biomarkers for differentiating disease stages and identifying molecular patterns associated with progression from T2DM to DPN and CF. **Methods:** The study included the following four groups: healthy controls, T2DM patients without complications, T2DM patients with DPN, and T2DM patients with CF. Expression profiles of five miRNAs (miR-19b-3p, miR-451a, miR-199a-3p, miR-146a-5p, and miR-93-5p) were quantified using qPCR. Inflammatory cytokine biomarkers including NLRP3, TNF-α, NF-κB, IL-1β, caspase-3, and Serpin E2 were measured using ELISA assays. **Results:** Distinct expression patterns of both miRNAs and inflammatory cytokine biomarkers were observed across diabetic neuropathy stages. Several miRNAs demonstrated significant dysregulation in DPN and CF compared with T2DM patients without complications. Correlation analyses revealed stage-specific patterns of interaction between inflammatory cytokines and miRNAs, indicating coordinated molecular alterations across different stages of diabetic neuropathic complications. **Conclusions:** These findings suggest that combining circulating miRNA and inflammatory marker profiles may improve the discrimination of CF from other diabetic neuropathic stages and may support clinical assessment when conventional diagnostic methods remain unclear. However, prospective longitudinal studies are required to determine their value for risk prediction and disease progression.

## 1. Introduction

Diabetes mellitus (DM) is one of the most common and fatal metabolic endocrine conditions in the world. Focusing on type 2 diabetes mellitus (T2DM), its prevalence is rising rapidly in non-industrialized populations. By 2025, it was estimated that three-quarters of the world’s 300 million adults with diabetes would be in non-industrialized countries, with almost a third in India and China alone. There is strong evidence that this epidemic has not only been triggered by social and economic development and urbanization, which is associated with general improvements in nutrition and longevity, but also with obesity, reduced physical exercise and other diabetogenic factors. Diabetic complications are categorized as macrovascular and microvascular. Macrovascular complications mainly include stroke and cardiovascular diseases whereas microvascular complications include diabetic retinopathy (affecting the retina of the eye), nephropathy (affecting the kidneys) and neuropathy (affecting the nerves of the body), especially resulting in the diabetic foot [1].

Approximately half of all diabetic patients, either pre-diabetes, type 1 or type 2 diabetes, will go on to develop diabetic neuropathy (DN) [2]. Symptoms of DN vary according to different stages of the disease. However, the commonality across all stages is the distal-to-proximal gradient of severity [2]. DN involves more sensory than motor impairments [2]. At the early stage, DN patients mainly experienced pain and hyperalgesia. As the disease progresses, patients experience numbness, muscle weakness, loss of balance, and foot ulcers [2]. DN patients experience poor quality of life and have high rates of ulceration and amputations [2].

To date, the treatment options for DN patients include intensive glycemic control, which slows the progression of the disease, pain relief drugs, anti-seizure medications, antidepressants, and foot care. However, a recent meta-analysis of DN studies has indicated that glycemic control does not benefit the majority of DN patients. Additionally, rapid glucose lowering can trigger neuropathic pain, which is known as treatment-induced neuropathy [2]. Thus, available treatments for DN are far from sufficient.

Charcot foot (CF) disease is a rare but severe complication of diabetes, which is associated with an increased risk of soft tissue infections, foot ulcers and amputations [3], as well as a high degree of morbidity and mortality [3]. The condition occurs most commonly in patients with diabetes suffering from severe diabetic peripheral neuropathy (DPN), often with coexisting sympathetic denervation, causing increased blood flow to the foot and increased bone resorption [3].

Inflammation is always present in CF disease and actively participates in the pathophysiology of the disease as is the case in most diabetic foot syndrome [3]. An excessive bone loss has been reported in CF; it is believed to be mediated by uncontrolled and excessive inflammation [3], leading to increased numbers of osteoclasts and their activity [3].

Despite advances in diabetic foot care, the early diagnosis of CF remains a major clinical challenge. In its early stages, CF often presents with non-specific clinical features such as swelling, erythema, and increased local temperature, which closely resemble cellulitis, osteomyelitis, or acute infection. Conventional radiographic imaging modalities such as X-ray frequently fail to detect early disease-related inflammatory and microstructural changes, as structural bone abnormalities typically appear only after significant and often irreversible destruction has occurred [3]. Although magnetic resonance imaging (MRI) can detect early inflammatory and other changes [4,5], its high cost, limited availability, and lack of routine use restrict its utility for widespread screening. Consequently, CF is frequently diagnosed at advanced stages, when deformity, ulceration, and risk of amputation are already established [3]. These limitations of delayed diagnosis and its consequences enforced us to try finding minimally invasive circulating biomarkers that can identify patients at high risk of developing CF at an early stage, particularly among individuals with DPN.

Increasing evidence indicates that microRNAs (miRNAs) are involved in the pathogenesis of DN [6]. miRNAs are non-coding RNA sequences composed of 18–24 nucleotides in length [7]. miRNAs bind to target mRNAs and induce translational repression and target mRNA decay [8]. Mature miRNAs can be released into circulation and body fluids. As they are protected by RNA-binding proteins and lipid-containing vesicles (micro-vesicles, exosomes, apoptotic bodies, and high-density lipoprotein), miRNAs show good stability and facilitate communication between cells or organs [9]. In the context of diabetes, hyperglycemia, hypoxia, and inflammation affect miRNA biogenesis and release. Consequently, these alterations in the miRNA profile are associated with multiple microvascular complications [10,11,12,13].

Of note, a significant portion of miRNAs was found to be specifically packaged into extracellular vesicles that express cell-type-specific proteins to mediate their different functions [14]. These findings support the potential applications of miRNAs in the diagnosis and therapy for DN [15]. In addition, it was hypothesized that they might possess the potential in diagnosing the pathogenesis of CF disease [3].

Therefore, the present study aimed to investigate the expression profiles of selected circulating miRNAs and inflammatory markers in patients with type 2 diabetes mellitus (T2DM) across different stages of diabetic neuropathic complications, including uncomplicated T2DM, DPN, and CF. The study further sought to evaluate their potential utility as minimally invasive biomarkers for discriminating between these disease-specific stages and identifying molecular signatures associated with advanced neuropathic complications. It is worth mentioning that identifying reliable circulating biomarkers capable of distinguishing different stages of diabetic neuropathic complications may contribute to improved clinical evaluation and facilitate earlier recognition of patients with more advanced disease involvement, thereby supporting more timely clinical management and helping to reduce the risk of irreversible foot deformities.

## 2. Subjects, Materials and Methods

### 2.1. Study Design and Participants

This cross-sectional case–control study was performed on 130 outpatients with (T2DM) and 43 healthy subjects serving as controls. T2DM patients were further subdivided into the following 3 different cohorts; first: diabetic patients without peripheral neuropathy [T2DM group, No. = 50], second: diabetic patients diagnosed with complication of peripheral neuropathy [DPN group, No. = 50] and third: diabetic patients diagnosed with DPN complicated to Charcot foot [CF group, No. = 30]. Patients in the CF group had clinically confirmed advanced Charcot neuro-osteoarthropathy and all exhibited established DPN, consistent with the recognized pathophysiology of the condition.

All patients were selected from outpatients Clinic of National Institute for Diabetes and Endocrinology (NIDE), Ahmed Maher Teaching Hospital, National Institute of Neuromotor System, Cairo, Egypt. This study was evaluated and approved by the research ethics committee of General Organization of Teaching Hospitals and Institutes with Ethics ID (IDE 00283). Written informed consent was obtained from all patients and healthy subjects that described the aim of the study and the procedures that would be required from them. The Declaration of Helsinki for Good Clinical Practice ethical guidelines were followed for this study. The privacy rights of human subjects were considered.

The following demographic and clinical data were recorded for each participant using self-made questionnaire: variables recorded for all participants include age, sex, duration of diabetes, body mass index (BMI) [calculated in Kg/m^2^ according to [16]], blood pressure [measurements were performed by trained technicians or nurses with a mercury sphygmomanometer; the first and fifth Korotk off sounds were recorded to represent the systolic blood pressure (SBP) and diastolic blood pressure (DBP). Two different measurements were obtained and averaged. Hypertension was considered if the SBP was G140 mm Hg or DBP was G90 mm Hg or taking medications of hypertension], nerve conduction study (NCS) [both motor nerve conduction velocity (MCV) and sensory nerve conduction velocity (SCV) studies were performed using an electromyography-evoked potential system], fasting plasma glucose level (FPG) [immediately determined utilizing glucose oxidase method according to [17]], glycated hemoglobin (HbA1c) [an ion exchange HPLC method was utilized for assaying HbA1c on G8 analyzer (Tosoh, Tokyo, Japan)], and lipid profile [serum total cholesterol was determined by the enzymatic method according to [18], triacylglycerol was assayed by peroxidase-coupled method according to [19], high-density lipoprotein cholesterol (HDL-c) was measured by enzymatic method according to [20], and low-density lipoprotein cholesterol (LDL-c) was measured according to [21]]. Sampling, reagent delivery, mixing, processing, calculating and printing were full automatically performed by BT3500 chemistry system (Biotecnica Instruments S.p.A., Rome, Italy).

### 2.2. Inclusion and Exclusion Criteria for Patients’ Cohort

The inclusion criteria were clinically diagnosed as T2DM patients according to recent standards [22], age between 35 and 65 years, HbA1c level ≥ 6.5% and diabetes duration > 2 years. T2DM patients were taking the same antidiabetic therapy (insulin).

DPN diagnosis followed guidelines recommended by the American Diabetes Association (ADA) and the Toronto Diabetic Neuropathy Consensus, considering the presence of clinical symptoms or signs and abnormalities in NCS [4,23].

Based on clinical evaluation and radiological findings CF was diagnosed according to the American Diabetes Association (ADA) and the American Podiatric Medical Association task force recommendations [4,5]. Patients typically presented with a swollen, erythematous foot, and radiological features consistent with Charcot neuroarthropathy, which may include bone destruction, fractures, and joint dislocations compared with the contralateral foot on plain X-ray imaging. In suspected early-stage cases, particularly in the presence of acute inflammatory signs such as redness, increased temperature, and tenderness of the foot, MRI was used to confirm the diagnosis, as it allows detection of bone marrow edema and inflammatory changes before the appearance of overt radiographic abnormalities.

Exclusion criteria included patients with type 1 diabetes, gestational diabetes, other forms of diabetes, neuropathies from other conditions, such as cervical and lumbar spine diseases, severe comorbidities like progressive malignant tumors, acute infections, severe renal impairment, or cardiovascular disease. Participants with abnormal renal or hepatic function were excluded to minimize potential confounding effects on circulating inflammatory biomarkers.

### 2.3. Inclusion and Exclusion Criteria for Healthy Control Participants

Healthy controls were recruited from hospital staff and community volunteers undergoing routine health evaluation and were age- and sex-matched to the patient groups. All control participants underwent clinical evaluation and laboratory screening to exclude DM, metabolic disorders, inflammatory diseases, and peripheral neuropathy.

The inclusion criteria for the healthy control group were adults aged ≥ 30 years, no prior diagnosis of diabetes mellitus (confirmed by normal FPG and HbA1c levels), no history or clinical symptoms suggestive of peripheral neuropathy, no clinical evidence of acute or chronic systemic disease, willingness to participate and provide written informed consent.

The exclusion criteria included Prediabetes or DM, any metabolic disorder (e.g., thyroid dysfunction, dyslipidemia requiring active treatment, or abnormal liver or kidney function tests), acute or chronic inflammatory, autoimmune, or infectious diseases, use of anti-inflammatory, immunosuppressive, or corticosteroid therapy and chronic renal, hepatic, cardiovascular, or malignant diseases.

### 2.4. Blood Sampling

Blood samples were collected after overnight fasting from all participants into three different types of vacutainer tubes. Blood from the first type (no additive) was centrifuged at 3000 rpm for 10 min. Serum was separated and divided into several aliquots; one aliquot was used to measure lipid profile at once and the other aliquots were stored at −80 °C for the determination of NOD-like receptor pyrin domain 3 (NLRP3), Tumor necrosis factor-α (TNF-α), caspase-3 (casp-3), Nuclear factor kappa-β (NF-kβ), Interleukin-1β (IL-1β) and Serpin peptidase inhibitor, clade E, member 2 (Serpin E2). Second part of collected blood was taken on 2 EDTA tubes, one for determination of HbA1c level and the second tube was centrifugated at 3500 for 5 min; plasma was separated and stored at −80 °C for later miRNA extraction. Hemolyzed samples were excluded. Third part of collected blood was taken on fluoride-containing tubes for determination of FPG.

### 2.5. Determination of Serum Biomarkers (NLRP3, TNF-α, Casp-3, NF-Kβ, IL-1β and Serpin E2)

Serum NLRP3, TNF-α, casp-3, NF-Kβ, IL-1β and Serpin E2 concentrations were quantitatively determined using available commercial ELISA Kits (Innova Biotech Co., Ltd., Beijing, China) (Cat. No.: In-Hu3841, In-Hu3936, In-Hu2595, In-Hu2637, In-Hu2151, In-Hu0619 respectively) according to the manufacturer’s instructions.

### 2.6. Fold Expression Level Estimation of Circulating miRNAs

Total RNA was extracted from plasma samples using the miRNeasy kit (Cat. No.: 217004) on fully automated system (QIAcube instruments, QIAGEN, Hilden, Germany) according to the manufacturer’s instructions. Reverse transcription was performed using a miRNA-specific reverse transcription kit, miRCURY LNA RT kit, (Cat. No.: 339340) according to the manufacturer’s protocol; reactions were incubated at 42 °C for 60 min followed by inactivation of the reaction by incubating at 95 °C for 5 min. Then, qPCR was conducted using specific primers for the selected miRNAs (miR-19b-3p, miR-451-a, miR-199a-3p, miR-146a-5p, and miR-93-5p) utilizing miRCURY LNA SYBR Green PCR kit (Cat. No.: 339347) on the Applied Biosystems Real Time PCR 96-well plate system [Stratagene Mx3000P™ real-time PCR system (Agilent Technologies, Santa Clara, CA, USA)]. Thermocycling conditions were as follows: initial heat activation at 95 °C for 2 min, followed by 40 cycles of denaturation at 95 °C for 10 s. and annealing/extension at 56 °C for 60 s. Relative miRNA expression levels were calculated using the 2^−ΔΔCt^ method [24], with U6 small nuclear RNA used as an internal reference for normalization.

### 2.7. Pre-Analytical Quality Control and Validation

To minimize pre-analytical variability in circulating miRNA measurements, all blood samples were processed using a standardized protocol. Peripheral venous blood was collected into appropriate collection tubes and centrifuged within one hour of collection. Plasma/serum separation was performed using a consistent centrifugation procedure by a single trained technician to ensure uniform handling. Samples were visually inspected for hemolysis, and any specimens showing red discoloration were excluded from further analysis. Aliquots designated for cytokine and miRNA measurements were immediately stored at −80 °C and thawed only once prior to RNA extraction and analysis to avoid RNA degradation. All equipment used all over the study was fully automated systems.

Regarding qPCR analysis, all samples were performed in duplicates to ensure reproducibility. In addition, melting curve analysis was performed for all qPCR reactions to confirm amplification specificity and exclude primer-dimer formation or nonspecific amplification products, ensuring reliable miRNA quantification.

These standardized pre-analytical procedures were applied to minimize technical variability and improve the reproducibility of circulating biomarker measurements.

### 2.8. Statistical Analysis

Statistical analysis was performed using IBM SPSS statistics software (version 22.0; IBM Corp., Armonk, NY, USA) and GraphPad Prism software (version 8; GraphPad Software, San Diego, CA, USA). Data were tested for normality using the Kolmogorov–Smirnov test; all variables showed normal distribution; therefore, parametric statistical tests were used in all analyses. Data were expressed as mean ± standard error of the mean (SEM). Unless otherwise stated, comparisons among multiple groups were performed using one-way analysis of variance (ANOVA), followed by Tukey’s post hoc test for multiple comparisons. Correlation analyses were conducted using the Pearson correlation coefficient for normally distributed variables. Receiver operating characteristic (ROC) curve analysis was performed to evaluate the diagnostic performance of individual microRNAs and inflammatory biomarkers in distinguishing between specified study groups. The area under the ROC curve (AUC), sensitivity, specificity, and optimal cutoff values were determined using the Youden index. All statistical tests were two-tailed, and a *p*-value < 0.05 was considered statistically significant. To evaluate the combined diagnostic performance of the investigated biomarkers, a binary logistic regression model was constructed. This model was used to compute predicted probability values, representing a combined diagnostic score based on the weighted contribution of the included biomarkers. These predicted probabilities were subsequently subjected to ROC curve analysis to assess the discriminative ability of the combined biomarker panel. The diagnostic accuracy of the combined model was evaluated using the AUC with its corresponding 95% confidence interval and *p*-value. Combined sensitivity and specificity were derived from the ROC curve using the Youden index to determine the optimal cutoff point. Improvements in AUC for the combined biomarker model compared with individual biomarkers were assessed descriptively.

## 3. Results

### 3.1. Demographic Characteristics of the Studied Cohorts

Regarding the demographic characteristics of the studied groups, BMI showed a statistically significant difference (*p* < 0.05) in all diabetic groups when compared to control groups. Similarly, statistically significant differences in SBP and DBP were found in the DPN and CF groups when compared to control and T2DM group (*p* < 0.05). In contrast, there are no statistically significant differences (*p* > 0.05) in terms of age and sex when compared to control group. The mean duration of diabetes was (3.60 ± 0.26, 16.62 ± 0.84, and 15.17 ± 0.98) respectively (Table 1).

### 3.2. Biochemical Characteristics Among the Control Group and the Diabetic Cohorts

Glycemic indices exhibited progressive increase across the studied diabetic groups; both FPG and HbA1c were significantly higher in DPN and CF groups when compared to T2DM group (*p* < 0.05). The highest FPG and HbA1c levels were recorded in CF group. In terms of lipid profile, total cholesterol and LDL-c were significantly decreased in CF group compared to diabetic groups (*p* < 0.05). Triglyceride was significantly decreased in CF group compared to DPN group (*p* < 0.05), while HDL-c was significantly decreased in DPN group compared to T2DM group and CF group (*p* < 0.05). NCS confirmed significant impairments in diabetic patients with neuropathy. Both SCV and MCV were significantly reduced in the DPN group compared with T2DM group (*p* < 0.05) (Table 2).

### 3.3. Cytokine Biomarkers and miRNA Profiles Among the Control Group and Different Diabetic Cohorts

**Figure 1:**
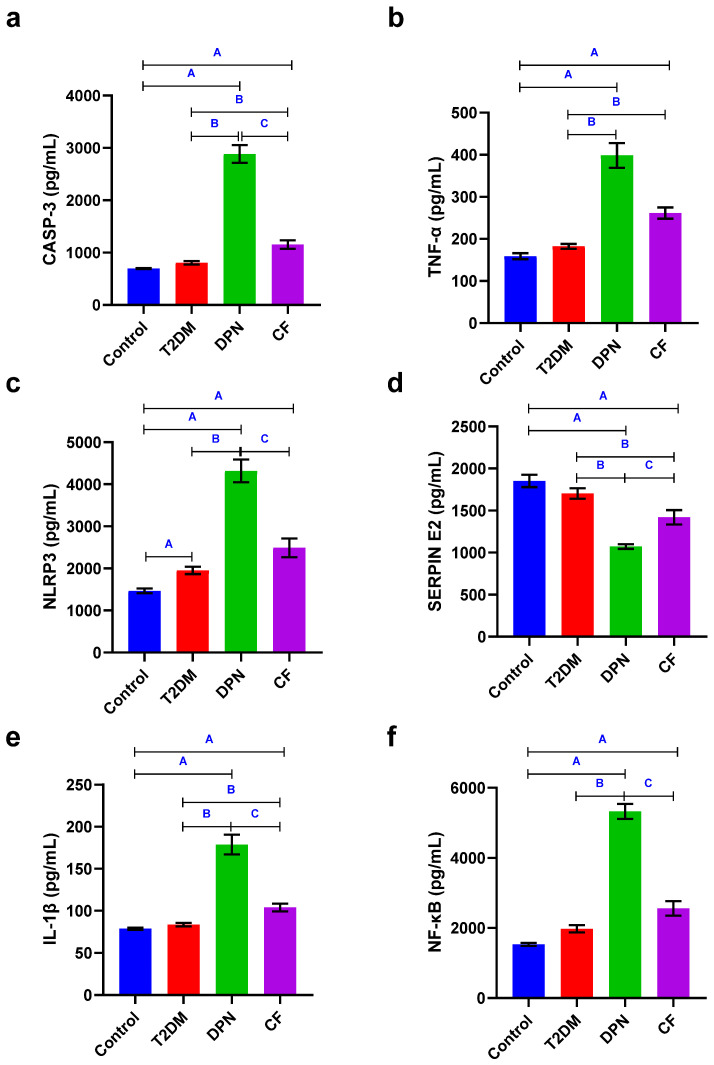
(**a**–**f**): cytokine biomarker patterns among the control group and different diabetic cohorts expressed as Mean ± SEM. A, B, C: *p* < 0.05 compared to the healthy control, T2DM, DPN groups respectively using one-way ANOVA followed by Tukey multiple comparison test. CASP-3: caspase-3; TNF-α: Tumor necrosis factor-α; IL-1β: Interleukin 1β; NF-kβ: Nuclear factor Kabba β; NLRP3: NOD-like receptor pyrin domain 3. Control: healthy control group. T2DM: type 2 diabetes mellitus patients without complications. DPN: diabetic peripheral neuropathy. CF: Charcot foot.

Regarding serum casp-3 and IL-1β levels, there was a significant increase in DPN group compared to control and T2DM groups (*p* < 0.05). In contrast, the CF group showed a significant decrease in these markers compared to the DPN group, while still exhibiting significantly higher levels than the control and T2DM groups (*p* < 0.05). Similarly, there was a significant increase in TNF-α in DPN group compared to control and T2DM groups (*p* < 0.05). Also, the CF group showed a significant increase in TNF-α as compared to control and T2DM groups (*p* < 0.05). Additionally, both NF-kβ and NLRP3 exhibit a significant increase in the DPN group when compared to control and T2DM groups (*p* < 0.05), whereas in CF group, there is a significant decrease when compared to DPN group (*p* < 0.05). However, no significant difference in NF-kβ and NLRP3 levels was noticed between the CF and T2DM groups (*p* > 0.05), although the observed increased level in CF group. In contrast, Serpin E2 level showed a significant decrease in the DPN group when compared to control and T2DM groups (*p* < 0.05) while in the CF group, it showed a significant increase when compared to DPN group (*p* < 0.05) and a significant decrease when compared to T2DM group (*p* < 0.05) (Table 3 and Figure 1a–f).

**Figure 2:**
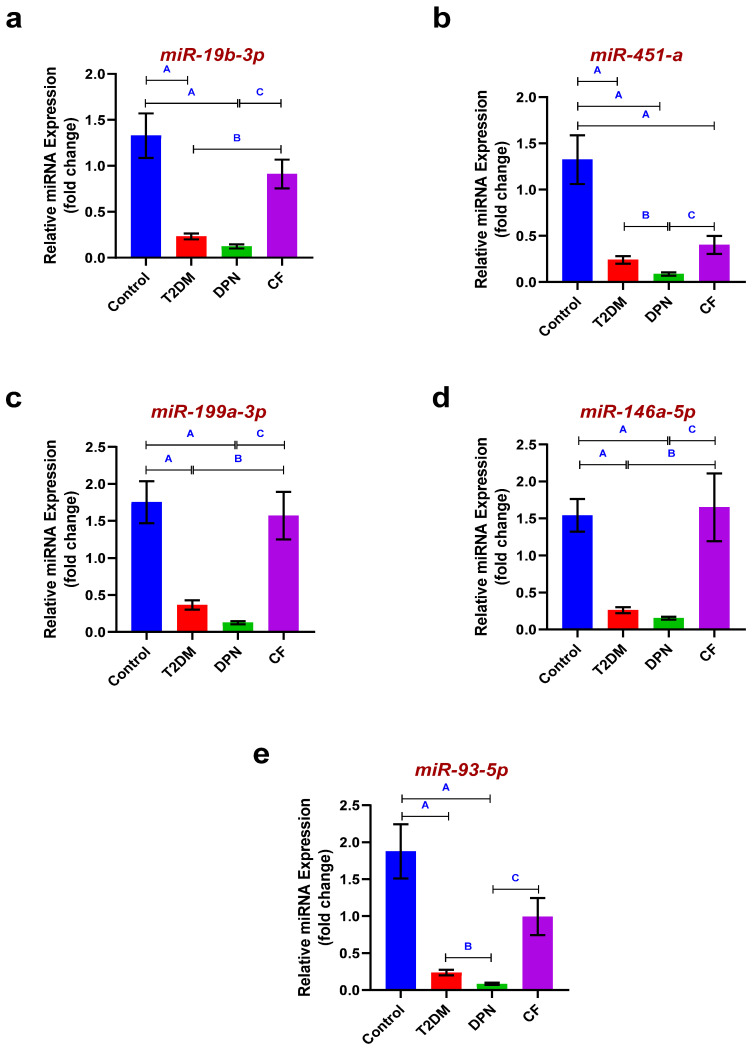
(**a**–**e**): miRNAs profile patterns among the control group and different diabetic cohorts expressed as Mean ± SEM of the miRNA fold expression. A, B, C: *p* < 0.05 compared to the control, T2DM, DPN groups respectively using one-way ANOVA followed by Tukey multiple comparison test. Control: healthy control group. T2DM: type 2 diabetes mellitus patients without complications. DPN: diabetic peripheral neuropathy. CF: Charcot foot.

Fold expression analyses of all analyzed circulating miRNAs revealed significant predominant downregulation in T2DM and DPN groups when compared to control group (*p* < 0.05). While miR-19b-3p was downregulated in DPN group compared to T2DM group insignificantly (*p* > 0.05), a significant increase in miR-19b-3p expression was observed in the CF group compared to both the DPN and T2DM groups (*p* < 0.05). miR-451-a expression was significantly downregulated in DPN group compared to both T2DM and CF groups (*p* < 0.05). However, although the CF group showed upregulated expression when compared to T2DM group, the difference was not statistically significant (*p* > 0.05). Regarding miR-199a-3p and miR-146a-5p, they were significantly upregulated in the CF group when compared to both T2DM and DPN groups (*p* < 0.05). However, although their expression was lower in the DPN group compared to the T2DM group, the differences were not statistically significant. miR-93-5p was significantly downregulated in DPN group compared to both T2DM and CF groups (*p* < 0.05). However, although its expression was higher in the CF group compared to the T2DM group, this upregulation failed to be statistically significant (Table 3 and Figure 2a–e).

### 3.4. Parametric Linear Correlations in Between the Investigated Parameters Among Different Diabetic Cohorts

Parametric linear correlation analysis was performed to explore the relationships between circulating inflammatory biomarkers, metabolic parameters, and miRNA expression levels within each diabetic cohort separately.

In T2DM group, caspase-3 exhibited positive correlation with FPG, IL-1β, NLRP3 and NF-kβ with and negative correlation with SCV. In DPN group caspase-3 shows negative with FPG and positive correlations with IL-1β and NF-kβ. In CF group, caspase-3 was positively correlated with NF-kβ. Regarding TNF-α, in DPN group, it showed positive correlations with IL-1β and NF-kβ and a negative correlation with Serpin E2. However, no significant correlations were observed in the T2DM and CF groups. In the T2DM group, IL-1β exhibited positive correlations with FPG and NF-kβ and a negative correlation with LDL-c. In DPN group, IL-1β has a positive correlation with NF-kβ. On contrary, IL-1β in the CF group showed a negative correlation with NLRP3. In T2DM group, NF-kβ exhibited positive correlation with HbA1c and negative correlations with cholesterol, LDL-c and SCV. In the CF group, NF-κB showed positive correlation with Serpin E2. NLRP3 exhibited positive correlation with FPG and negative correlation with Serpin E2 in T2DM group while showing positive correlations with BMI and HDL-c in the DPN group with no detected significant correlations in the CF group. Serpin E2 showed negative correlation with TG in the DPN group and positive correlation with BMI in the CF group with no observed significant correlations in T2DM group.

Importantly, strong positive intercorrelations were consistently observed among the measured circulating miRNAs across diabetic cohorts, with the highest correlation coefficients generally observed in CF patients. Several miRNAs also exhibited significant correlations with lipid parameters, particularly LDL-cholesterol. Detailed correlation coefficients and significance levels are presented in Table 4.

### 3.5. ROC Curve Analysis Evaluating the Discriminative Performance for Individual and Combined Biomarkers: NLRP3, Serpin E2 and the Circulating miRNAs Under Two Clinical Comparisons; T2DM vs. DPN Patients (Figure 3A,C) as Well as DPN vs. CF Patients (Figure 3B,D and Figure 4A–C)

**Figure 3:**
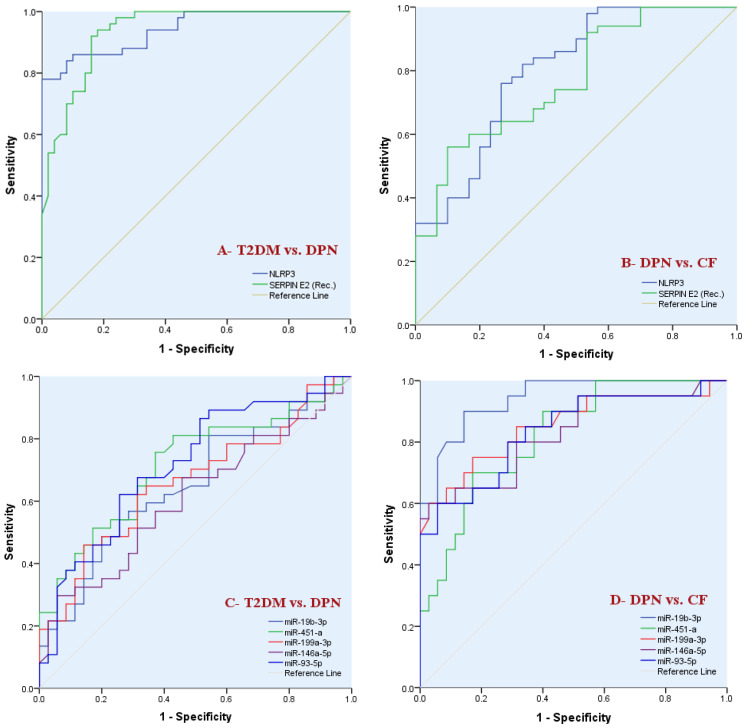
ROC curve analyses evaluating discriminative performance of individual inflammatory biomarkers (NLRP3 and SERBIN E2) and circulating miRNAs under the following 2 clinical comparisons: T2DM patients without peripheral neuropathy vs. DPN cases (**A**,**C**), and DPN patients vs. CF patients (**B**,**D**). Diagnostic accuracy is expressed as the area under the curve (AUC). Corresponding AUC values and *p*-values are presented in Table 5. (**A**): the 2 investigated cytokines showed comparable excellent discriminative ability differentiating DPN cases from T2DM subjects without peripheral neuropathy. (**B**): although the 2 biomarkers demonstrated good discriminative utility in identifying CF cases out of those having DPN, NLRP3 exhibited superior discrimination. (**C**): all investigated miRNAs except for miR-146a-5p demonstrated comparable good differentiating ability. (**D**): all investigated miRNAs revealed good classifying accuracy, with miR-19b-3p showing excellent ability to differentiate CF cases from all patients having DPN.

**Figure 4:**
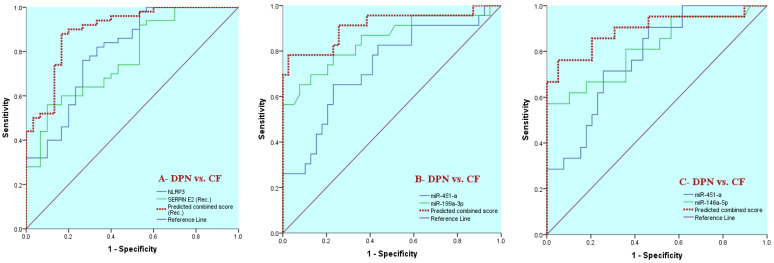
ROC curve analyses evaluating discriminative performance of combined biomarker panels in differentiating CF patients from all cases with DPN. Predicted probability scores derived from binary logistic regression models for combined inflammatory biomarkers (NLRP3 and SERBIN E2) (**A**) and selected miRNA pairs (miR-451-a with miR-199a-3p and miR-451-a with miR-146a-5p) (**B**,**C**) were subjected to ROC analysis. Diagnostic accuracy is expressed as the area under the curve (AUC). *p*-value and AUC-combined results were shown in Table 5 under “Predicted combined panel score” category with corresponding cut-off-combined value which discriminates efficiently between CF patients and ordinary DPN subjects. Combined biomarker panels demonstrated improved discriminative accuracy compared with individual biomarkers.

As represented in Table 5, ROC analysis was employed to evaluate the discriminative ability of inflammatory biomarkers (NLRP3 and Serpin E2) and circulating miRNAs under two clinical comparisons as follows: T2DM without neuropathy vs. DPN cases (No. = 100) (Figure 3A,C) as well as DPN patients vs. CF patients (No. = 80) (Figure 3B,D and Figure 4A–C).

For differentiation between T2DM and DPN patients, the analysis revealed that both NLRP3 and Serpin E2 demonstrated excellent discriminative ability, with AUC values of 0.94 for each marker (*p* < 0.001), indicating high accuracy in identifying DPN. On the other hand, individual circulating miRNAs showed modest to good discriminative ability in this comparison. The expression levels of only four investigated miRNAs (miR-19b-3p, miR-451-a, miR-199a-3p and miR-93-5p) succeeded well to discriminate between DPN against T2DM patients [AUC = 0.65, *p* = 0.018 (95% CI: 0.52–0.78), sensitivity: 100%, specificity: 88.6%, AUC = 0.72, *p* = 0.001 (95% CI: 0.59–0.83), sensitivity: 75.7%, specificity: 62.9%, AUC = 0.66, *p* = 0.012 (95% CI: 0.53–0.78), sensitivity: 45.9%, specificity: 85.7%, and AUC = 0.72, *p* = 0.001 (95% CI: 0.59–0.83), sensitivity: 62.2%, specificity: 74.3%] respectively.

When comparing DPN patients with CF patients, both inflammatory biomarkers demonstrated good diagnostic accuracy, with NLRP3 showing superior performance (AUC = 0.80) compared with Serpin E2 (AUC = 0.77) (*p* < 0.001). The same was observed for the expression levels of all investigated circulating miRNAs which exhibited good to excellent discriminative ability in distinguishing CF from DPN patients, with miR-19b-3p showing outstanding performance (AUC = 0.94), followed by miR-199a-3p, miR-146a-5p, miR-93-5p, and miR-451-a (AUC range 0.82–0.85).

To further assess whether combining biomarkers enhances discriminative performance for identifying CF among patients with DPN, binary logistic regression models were constructed, and predicted probability scores were generated for selected biomarker combinations. Combined analysis of NLRP3 and Serpin E2 improved the overall discriminative performance compared with either biomarker alone (AUC = 0.90). Similarly, combined miRNA panels, particularly miR-451-a with miR-199a-3p and miR-451-a with miR-146a-5p, yielded higher AUC values (0.91 and 0.90, respectively), with improved sensitivity and specificity for differentiating CF from DPN patients (Table 5; Figure 4). These findings indicate that combined biomarker panels provide superior discriminative accuracy compared with individual markers in identifying CF among patients with DPN.

## 4. Discussion

CF remains a challenging diabetic complication due to the lack of reliable tools for early identification, as conventional clinical and radiological approaches often detect the disease only after irreversible damage has occurred [3]. Currently, the treatment options for DPN are limited to intensive glycemic control, pain relief drugs and foot care which often provide limited benefit to most patients [2]. In this context, the present study investigated circulating inflammatory markers and miRNAs across progressive stages of diabetic neuropathy (uncomplicated T2DM, DPN and CF) to address this unachievable clinical need. Our aim was to explore the potential utility of these biomarkers, individually and in combination, as adjunctive indicators capable of discriminating between different stages of diabetic complications, particularly in distinguishing Charcot foot from other neuropathic conditions in high-risk diabetic patients.

In the present study, efforts were made to minimize potential confounding factors that could influence circulating inflammatory markers and microRNA levels. The study groups were matched for age and sex, while participants with abnormal renal or hepatic function were excluded based on laboratory screening. In addition, all diabetic participants were receiving insulin therapy, reducing variability related to antidiabetic medications. Although diabetes duration differed between uncomplicated T2DM and complication groups, no significant difference was observed between the DPN and CF groups, which represent the primary comparative groups of interest in the present study. These precautions were taken to ensure that the observed biomarker alterations were more likely related to disease stage rather than external clinical factors.

In this study, control subjects were selected to age- and sex-matched diabetic patients to ensure accurate comparisons and minimize the potential influence of age and gender differences. However, we were unable to achieve equal BMI distribution between diabetic and control groups. This imbalance likely reflects the high prevalence of obesity as a major risk factor among individuals with T2DM. Nevertheless, there is no current evidence to suggest that differences in BMI significantly affect the parameters investigated in this study. Although BMI was higher in diabetic patients compared with healthy controls, no significant differences were observed among the diabetic subgroups. Therefore, the biomarker differences observed between DPN and CF groups are unlikely to be explained solely by differences in adiposity. Additionally, among diabetic groups, there were no statistical differences concerning age or sex, allowing more accurate results. Regarding the duration of diabetes, the mean disease duration among all T2DM patients was approximately 15 years compared to controls. It is well-known that increased disease duration emerges as an independent risk factor for DPN [25]. As expected, patients with diabetic complications (DPN and CF) demonstrated a significantly longer duration of diabetes compared with the uncomplicated T2DM group, reflecting the well-established relationship between prolonged disease duration and the development of neuropathic complications [25]. In the same line, Bansod et al. reported that duration of diabetes contributes to the development of Charcot neuroarthropathy (CN) in diabetic patients [26]. Similarly, hypertension appeared to be a significant risk factor for the development of PDN. This may be owed to its direct damaging effect on the small blood vessels that supply nerves; previously, Yashendra et al. reported that hypertension appears to be the leading modifiable risk factor for the development of DN [27].

Regarding glycemic indices (FPG and HbA1c), a persistent state of hyperglycemia contributes to a marked dyslipidemia in DPN patients [28]. The etiology and pathogenesis of DPN are still inconclusive but are currently thought to be mainly related to a series of pathophysiological processes caused by hyperglycemia, dyslipidemia, and insulin resistance [28]. Abnormal glucose-lipid and insulin resistance and its sequelae cause alterations in mitochondrial function, inflammation, oxidative stress, specific gene transcription, and expression, ultimately leading to neuronal–glial cell damage [28]. The highest level of HbA1c was found in CF patients, which is in accordance with Himani et al., who reported that persistent exposure to hyperglycemia and accompanying complications, such as sensory neuropathy, extensively influences CN development. High HbA1c levels indicate poor glycemic control, contributing to vascular changes and neuropathy that underlie CN [26]. Overall, all investigated clinical data clearly indicate progressive metabolic decline among diabetic patients, characterized by deteriorating glycemic control, lipid profile alterations, and significant nerve conduction deficits in those with peripheral neuropathy, with the most severe changes observed in patients with CF.

DPN, a severe condition caused by DM, is a complication that affects a large number of people. This occurs when nerve injury causes painful and incapacitating symptoms [29]. Research has highlighted how crucial cytokines, proteins involved in inflammation, are in developing DPN [29]. These biomarkers can be linked to injury in the peripheral nerves, shedding light on how inflammation plays a role in this condition. These cytokines are produced in response to hyperglycemia and other metabolic disturbances associated with diabetes [29]. In this study, we investigated the role of certain proteins and cytokines as part of the pathway involved in the development of DPN and CF, which represents a severe form of DPN.

Regarding NF-kβ, it is a crucial component in various mechanisms naming immunological responses, cell death, viral replication and cancer [30]. The induction of NF-kβ can be regarded as a cellular reaction to stress, which can be induced by several mechanisms including bacterial and viral infections, cytokines, and interaction of antigen receptors [31]. NF-kβ comprises many subunits, namely p65 (RelA), RelB, c-Rel, p105/p50 (NFκB1), and p100/P52 (NFκB2) [32]. NF-kβ functions as an inflammatory modulator, and there exists a robust correlation between inflammation and the progression of diseases [33]. The results of our study revealed that serum level of NF-kβ was elevated in DPN group compared to that with T2DM group. A study by Y. Yang et al. reported that the NF-kβ in the advancement of DN can be attributed to its function in orchestrating inflammation [33]. Ischemic/reperfusion damage increases inflammation, which can be ascribed to the NF-kβ [33]. The activation of signaling pathways such as NF-kβ leads to increased expression of pro-inflammatory cytokines. This cascade contributes to oxidative stress and neuronal damage [29]. Inflammation and oxidative damage are key factors in the progression of DN. So, intensified inflammation increases the production of NF-kβ, which disturbs the balance of antioxidants by reducing the activity of Nrf2 [34].

Oxidative/nitrosative stress in addition to hyperglycemic conditions further activates the NF-kβ leading to the generation of pro-inflammatory cytokines, TNF-α, IL-6, and IL-1β, thereby promoting inflammation and apoptosis leading to the development of DN [1]. Activation of NF-kβ leads to increased expression of TNF-α and other inflammatory mediators, resulting in cellular injury, oxidative stress, and further nerve damage [29].

TNF-α is a pro-inflammatory cytokine implicated in the development and progression of DN [35]. Peripheral nerve degeneration is facilitated by elevated TNF-α levels, which are linked to increased oxidative stress and neuronal damage [35]. TNF-α levels and DPN are significantly correlated by a meta-analysis, suggesting that this protein may be a promising therapeutic target for the treatment of DN [35]. Our results showed increased level of TNF-α in DPN group compared to those with T2DM group; this is in accordance with previous studies which observed an elevated level of TNF-α and documented its correlation with reduced NCV and increased pain symptoms in DN patients [36,37]. Research shows that blocking TNF-α can improve nerve function and alleviate pain [36,37].

IL-1β is another critical cytokine that contributes to the inflammatory response in DN. It is released following nerve injury and acts synergistically with TNF-α to amplify the inflammatory response [37]. Similar to TNF-α, IL-1β can induce hyperexcitability in neurons by enhancing the phosphorylation of NMDA receptors, which are crucial for pain transmission. This mechanism contributes to the development and maintenance of neuropathic pain [38]. IL-1β also promotes microglial activation, leading to further release of inflammatory mediators that perpetuate pain signaling pathways [36]. So, IL-1β have the same increased level in DPN group compared to those with T2DM group. It is reported that, in the CNS, activated microglia releases pro-inflammatory cytokines like TNF-α and IL-1β upon exposure to hyperglycemia or oxidative stress. This release further exacerbates neuronal damage by promoting a neuroinflammatory environment [36]. The presence of these cytokines is linked to increased neuronal excitability and pain perception, contributing to the symptoms of DN [39].

Serum levels of TNF-α, IL-1β, and IL-6 are considerably greater in DN patients than in healthy people, according to clinical research. Reduced sensory NCV, a marker of nerve fiber injury and dysfunction, is greatly linked to the increased levels of these inflammatory cytokines [29]. Additionally, treatment approaches focusing on these cytokines promise to improve nerve function and lower neuropathic pain [29,39].

CN, also known as neuropathic osteoarthropathy, CF, or simply Charcot joint, is a rare, weakening condition primarily affecting individuals with DM. This disorder is characterized by progressive neuropathy, deformity, and joint destruction, leading to substantial morbidity and functional impairment [5]. In diabetes, CN signifies a clinical presentation indicated by impaired proprioception, autonomic dysfunction, and profound sensory neuropathy. It classically manifests in the lower extremities, particularly the foot and ankle, but other joints are also involved [40].

The occurrence and progression of diabetic CF results from the synergistic interaction between neuropathy and inflammation, which collectively impair the stability of the bone joint structure and trigger pathological processes. Neuropathy affects bone integrity, joint sensation, and blood flow regulation in the distal extremities, leading to the loss of protective sensation and autonomic nerve dysfunction. Inflammation, on the other hand, exacerbates tissue damage through mechanisms such as immune cell activation and cytokine release [41]. Acute local inflammation of the foot and ankle can lead to varying degrees of destruction, subluxation, dislocation, and foot deformities, including the typical “rocker-bottom” deformity caused by midfoot collapse [5].

Our result showed increased levels of TNF-α, IL-1β and NF-kβ in CF group when compared to T2DM group. Characteristic elevated levels of pro-inflammatory cytokines, such as IL-6, TNF-α, IL-1β, and monocyte chemoattractant protein-1, illustrates a state of chronic low-grade inflammation, impairing immune function and improper execution of lymphocytes, macrophages, and neutrophils [26]. These cells are vital for tissue repair and defense against pathogens, leading to increased susceptibility to infections, impaired wound healing, and lowered natural immune response. Due to compromised immunity in diabetic individuals, conditions such as diabetic foot infections are of significant concern in CN [26]. Diabetes causes an imbalance between bone formation and resorption, leading to osteopenia. Due to the amplified bone resorption process, bones are more susceptible to fractures as bone density is decreased. Hormonal imbalances due to diabetes lead to decreased insulin-like growth factor 1 levels, weakening the bones and making joints more susceptible to damage [26]. At the molecular and cellular level, activation of the receptors for advanced glycation end products and NF-kβ signaling advances the development of neurovascular dysfunction, neuropathy, and upregulation of pro-inflammatory cytokines [26]. Another study revealed that when comparing patients with CF versus patients with T2DM and without DM, the inflammatory markers’ tendency is to show increased levels of IL-6, IL-1β, TNF-α [42].

In contrast, when comparing our results in CF group with DPN group, there is a decrease in TNF-α, IL-1β and NF-kβ. This may be due to the fact that, after some months, the inflammation settles and this is closely associated with the end of the lytic–destruction phase [43]. Additionally, as previously reported, in advanced CF patients, although the fracture is associated with pain and this may lead to splinting of the bone, the rise in proinflammatory cytokines in this case is usually relatively short-lived [5].

Caspase-3 is one of the members of the family of caspases (cysteine-aspartic proteases), which are proteolytic enzymes known for their key roles in controlling inflammation [44]. It is also one of the key indicators of apoptosis, known as an executioner caspase because of its potent role in coordinating the destruction of cellular structures such as DNA fragmentation or degradation of cytoskeletal proteins [45]. Our results showed a significant increase in caspase-3 in DPN group compared to T2DM group. The overexpression of apoptosis-related proteins, BAX, BCL2, and caspase-3 in the sciatic nerve is reported in streptozotocin-induced DPN in rats [46]. Wu et al. elucidated that sciatic nerve injury induced by the chronic constriction injury model not only initiates chronic neuropathic pain but also increases the expression of caspase-3 in the spinal cord and caspase-3-dependent apoptosis of dorsal horn neurons, which is associated with up-regulation of growth-associated protein 43 expression [47].

Because of its important role in inflammatory pain, it was expected that caspase-3 is elevated in CF when compared to T2DM due to its role in apoptosis, which is a feature of the inflammatory process in this condition [48]. However, in contrast to this expectation, its level was reduced in CF group compared to DPN group. This may be explained in part as, in people with diabetes mellitus, the CF is a specific manifestation of peripheral neuropathy that may involve autonomic neuropathy with high blood flow to the foot, leading to increased bone resorption. It may also involve peripheral somatic polyneuropathy with loss of protective sensation and high risk of unrecognized acute or chronic minor trauma. In both cases, there is excess local inflammatory response to foot injury, resulting in local osteoporosis. Notably, CF may involve acute and chronic phases. The acute phase is characterized by local erythema, edema, and marked temperature elevation, while pain is not a prominent symptom. In chronic phase, signs of inflammation gradually recede and deformities may develop, increasing the risk of foot ulceration [49].

Inflammasomes are complexes of muti-domain proteins including pro-caspase-1, the activation of which releases caspase-1, which, in turn, cleaves pro-IL-1β into IL-1β, causing abnormal pain. NLRP3 is a key molecule within the inflammasome, which can be activated by thioredoxin-interacting protein and has attracted much attention in the field of pain. Upregulation of NLRP3 inflammasome in the sciatic nerve and dorsal root ganglion is reportedly implicated in the pathogenesis and progression of DPN [50]. Excessive NLRP3 inflammasome activation leads to maturation and promotes the release of pro-inflammatory mediator IL-1β in diabetic complications including DPN [50] as well as in the sciatic nerve of diabetic rats [50]. Inflammatory reactions caused by IL-1β release from the NLRP3 inflammasome lead to the advancement of neuropathic pain [50]. The inflammatory response is one of the essential pathologic features that link to the onset and progression of DPN. Therefore, NLRP3 inflammasome-mediated pyroptosis and inflammation are involved in the progression of DPN [50]. As the inflammasome-driven inflammation contributes to a wide range of inflammatory reactions, targeting the NLRP3 inflammasome pathway is vital for the treatment of inflammation-associated diseases such as pulmonary disease, asthma, coronavirus disease 2019 and DPN [50]. Hence, a promising therapeutic approach in management of DPN is trying to decrease the NLRP3 inflammasome activation [50]. Previous works have demonstrated that Schwann cell loss occurs in both patients with clinically established DPN and in experimental models of diabetes. In contrast, interventions that preserve Schwann cell function have been shown to attenuate the progression of DPN [50]. Accordingly, targeting inflammasome activation and Schwann cell apoptosis may represent a potential therapeutic strategy for the management of DPN.

NF-kβ pathway modulates cell proliferation and differentiation, morphogenesis, apoptosis and inflammasome activation, thereby triggering inflammation via enhancing the release of several cytokines, chemokines and adhesion molecules. Several stimuli trigger NF-kβ playing a regulatory role in the inflammatory response, stress response, pyroptosis and apoptosis. NF-kβ/NLRP3 inflammasome activation is a key factor in the development of DM. The correlation between NLRP3 inflammasome activation and pyroptosis in DPN offered a theoretical foundation for regulating pyroptosis and exerting a protective effect by inhibiting the NF-kβ/NLRP3 signaling axis [50].

NLRP3 inflammasomes have been profoundly attributed to the pathogenic mechanisms that induce T2DM and its related complications [50]. Ding et al. revealed that NLRP3 inflammasome modulates endoplasmic reticulum stress, which regulates glucose tolerance, insulin resistance, inflammation and apoptosis in adipose tissue in DM [50].

Fortunately, our results came supporting all above-mentioned explanations about the elevated levels of NLRP3 in DPN group compared to T2DM group.

Regarding NLRP3 in CF, they exhibit no significant difference when compared with T2DM group, but when compared to DPN group, there is a significant decrease in NLRP3 level, which may be due to the fact that NLRP3 is an inflammatory protein and has a pathway with IL-1β and NF-kβ [50], as they exhibit the same decreased level as shown in both inflammatory markers, in which they decreased in CF when compared to DPN.

Serpin peptidase inhibitor, clade E, member 2 (Serpin E2), is an extracellular matrix protein but can also be distributed in the cell membrane and cytoplasm. The molecular weight of the serpin E2 protein is in the range 45–50 kDa, and it is encoded by a gene on human chromosome 2q99-q35. Serpin E2 belongs to the nexin protease family, and it is also known as protease nexin-1. Nevertheless, serpin E2 is expressed in multiple organs and by various cell types, including macrophages, astrocytes, smooth muscle cells, vascular cells, and platelets [51].

In the present study, circulating Serpin E2 levels were significantly reduced in patients with DPN compared with uncomplicated T2DM subjects. This finding is consistent with the observations of Ying et al., who reported downregulation of Serpin E2 in T2DM patients with neuropathy [52]. Serpin E2 is known to regulate tissue plasminogen activator activity, which has been implicated in hypoxia–ischemia-related injury and excitotoxicity processes that contribute to diabetic nerve damage through proteolytic and receptor-mediated pathways [53]. Interestingly, an opposite expression pattern was observed in patients with CF, in whom Serpin E2 levels were significantly increased compared with the T2DM group. Although the mechanistic basis of this finding remains unclear, it may reflect the distinct pathophysiological environment of CN, which is characterized by advanced neuropathy, chronic inflammatory pattern, progressive bone and joint destruction, and marked tissue remodeling [3]. In this context, the elevated Serpin E2 levels observed in CF may represent a compensatory or disease-stage-specific response related to chronic inflammation and structural damage rather than neuropathy alone. Because very limited information is available regarding Serpin E2 expression in CF, further studies are urgently needed to clarify its role and to determine whether its differential regulation can aid in distinguishing advanced neuropathic complications from earlier stages of diabetic neuropathy.

The diagnosis of DN relies on a combination of clinical evaluation and multiple qualitative and quantitative investigations. In addition to assessing symptoms such as muscle weakness, numbness, and impaired reflexes, patients commonly undergo laboratory tests to evaluate glycemic control, vitamin status, and immune function, as well as imaging modalities including MRI, CT scans, and nerve or muscle ultrasound. Neurophysiological assessments, such as electromyography and NCV tests, are frequently used, while invasive procedures including nerve or skin biopsy are reserved for selected cases [2]. Despite their clinical value, these approaches are often costly, time-consuming, invasive, or limited in their ability to detect early or progressive neuropathic changes. This has highlighted the need for non-invasive molecular biomarkers that may support the detection and differentiation of diabetic neuropathic complications. In this context, circulating miRNAs have gained increasing attention. Altered miRNA expression profiles have been reported in diabetic patients across various stages of disease [1,2]. Because miRNAs are detectable in biological fluids and exhibit remarkable stability, they represent promising candidates for use as diagnostic or prognostic biomarkers and, potentially, therapeutic targets [2]. Accordingly, the present study evaluated selected circulating miRNAs to explore their association with different stages of DN and CF, aiming to assess their potential utility as minimally invasive biomarkers that may aid in identifying patients at higher risk of disease progression.

miR-19 belongs to the miR17-92 family [54] and contains two mature forms (a and b), each containing two 3-p and 5-p types. Among different miR-19 subtypes, miR-19b-3p has the highest expression in peripheral blood [55]. It has multiple essential roles, including regulatory effects on Toll-like receptor (TLR2), TLR4, TNF-α, IL-6, IL-8, IL-1β, Matrix metalloproteinase through NF-kβ, Mitogen-activated protein kinase (MAPK), TNF receptor-associated factor (TRAF), Interleukin-1 receptor-associated kinase (IRAK), Janus kinase (JAK)/signal transducer and activator of transcription (STAT) signaling pathways, and the expression of Phosphoinositide 3-kinase (PI3K), and Protein kinase B (PKB or AKT), leading to inhibition of apoptosis and induction of Schwann cells growth [55]. The findings of the present study are consistent with previous reports demonstrating reduced expression of miR-19b-3p in the peripheral blood of patients with DPN compared with healthy controls and T2DM patients without complications. This observation agrees with Rajabinejad et al., who reported a significant downregulation of miR-19b-3p in patients with DN relative to non-neuropathic diabetic patients and healthy individuals [55]. miR-19b-3p has been shown to play a regulatory role in inflammatory signaling pathways, particularly influencing the expression of TNF-α, IL-1β, and NF-κB [55]. In line with that fact, the downregulation of miR-19b-3p observed in the DPN group in our study was accompanied by increased levels of these inflammatory markers, supporting a potential link between miR-19b-3p dysregulation and enhanced inflammatory activity in the pathogenesis of DPN.

In contrast, miR-19b-3p expression was significantly upregulated in the CF group compared with both the DPN and T2DM groups. This finding is consistent with the report by Jennifer et al., who demonstrated elevated miR-19b-3p levels in patients with CF [3], suggesting a possible potential association with advanced CN. Importantly, this observation aligns closely with our inflammatory profile, as TNF-α levels were found to be reduced in the CF group compared with the DPN group. Previous studies have shown that miR-19b-3p can inhibit TNF-α–induced endothelial cell apoptosis through an Apaf-1/caspase-dependent pathway [56]. Taken together, the concomitant upregulation of miR-19b-3p and reduction in TNF-α observed in our CF patients may reflect a stage-specific regulatory or compensatory response aimed at modulating inflammatory injury and tissue remodeling during advanced disease stages. These findings further support the idea that miR-19b-3p participates in inflammation-related pathways relevant to CF progression and highlight the importance of interpreting miRNA alterations within the broader inflammatory context [55] rather than as isolated molecular changes.

The miR-144/451 cluster, located on chromosome 17q11.2, represents a unique and evolutionarily conserved genomic structure in vertebrates. This cluster consists of the following three key members: miR-144, miR-451a (mature sequence): 5′-AAACCGUUACCAUUACUGAGUU-3′, commonly referred to as miR-451 in the literature, and miR-451b (mature sequence): 5′-UAGCAAGAGAACCAUUACCAUU-3′. These miRNAs, while sharing genomic proximity, exhibit distinct characteristics in their sequences, biogenesis pathways, and biological functions. Expression of miR-451a is particularly prominent in erythroid cells and is crucial for erythropoiesis. miR-451a has been extensively documented for its roles in hematopoietic differentiation, tumor suppression, and inflammatory regulation [57]. In the present study, miR-451a expression was significantly downregulated in the DPN group compared with both T2DM and healthy control groups, a finding that coincided with increased inflammatory activity as shown by increased levels of TNF-α, IL-1β, and NF-κB. This observation is consistent with the proposed role of miR-451a as a negative regulator of inflammatory signaling pathways [57]. In contrast, miR-451a expression was markedly upregulated in the CF group compared with both DPN and T2DM groups. This finding aligns with the report by Jennifer et al., who demonstrated elevated miR-451a levels in patients with CF and suggested its potential involvement in modulating NF-κB-mediated inflammatory responses [3]. Notably, the upregulation of miR-451a in the CF group in our study was accompanied by a significant reduction in NF-κB levels compared with the DPN group, supporting a possible stage-specific or compensatory regulatory role of miR-451a in advanced neuropathic disease. Together, these findings again suggest that alterations in miR-451a expression may reflect changes in inflammatory regulation across different stages of DN and CF progression.

miR-199 is a highly conserved miRNA family, with the following two members: miR-199a and miR-199b. Currently, there are two types of pre-miRNAs: pre-miR-199a-1 and pre-miR-199a-2, derived from chromosomes 19 and 1, respectively. After the cleavage of miR-199a by Dicer, the following two kinds of mature miRNAs could be obtained from the 5′ arm and 3′ arm, respectively: miR-199a-5p and miR-199a-3p [58]. In the present study, miR-199a-3p expression was reduced in patients with DPN compared with the T2DM group (non-significant) and healthy controls (significant). This finding is consistent with previous work by Wang et al., who reported downregulation of miR-199a-3p in patients with T2DM complicated by macrovascular and microvascular lesions [59], suggesting a link between reduced miR-199a-3p expression and diabetic vascular and neural complications.

In contrast to our findings, Ying et al. reported that overexpression of miR-199a-3p inhibited Serpin E2 expression in patients with DPN [52]. In our study, both miR-199a-3p and Serpin E2 exhibited a similar downregulated pattern in the DPN group, indicating a discrepancy with previously reported inverse relationships. This inconsistency may reflect population-specific differences, disease heterogeneity, or variations in disease stage and metabolic control. Further larger studies, particularly in Egyptian diabetic cohorts, are eligible to clarify the regulatory relationship between miR-199a-3p and Serpin E2 in DN.

Notably, miR-199a-3p expression was significantly upregulated in the CF group compared with both the DPN and T2DM groups. This finding may be partially explained by the metabolic characteristics of the CF group in the present study, which exhibited the highest HbA1c levels. Supporting this interpretation, Ying et al. reported a positive association between elevated HbA1c levels and increased miR-199a-3p expression [52]. Together, these findings suggest that miR-199a-3p expression may vary across different stages of diabetic complications and may be influenced by glycemic status, highlighting its potential relevance in distinguishing advanced neuropathic conditions such as CF.

miRNA 146a, which is encoded by the MIR146A gene located on chromosome 5q33.3, is a small non-coding RNA [60]. Our result showed a significant downregulation of miR-146a-5p in DPN group if compared to T2DM and control group. miR-146a is one of the most important miRNAs that is dysregulated in diabetic conditions. miR-146a contributes to the adaptive and innate immune responses, and its role as an anti-inflammatory miRNA in the pathogenesis of DN has been established in mice models [1]. Also, the levels of miR-146a were found to be downregulated under hyperglycemic conditions [1]. miRNA-146 located in circulating mononuclear cells were established to modulate inflammatory response in DPN [61,62].

A previous report demonstrated that miR-146a and NF-kβ may be involved in the pathophysiological mechanism of DPN [61]. Our results came supporting; there is a significant increase in NF-kβ in DPN group at the same time where miR-146a is significantly downregulated. As reported previously, miR-146 expression was highly associated with inflammatory cells [63]. This had also been manifested on animals where downregulating miR-146a results in overproduction of TNF-α and IL-1β as well as loss of NF-kβ inhibition in the sciatic nerve of DPN mice [64]. Our results came supporting all these published data showing simultaneous increase in all inflammatory cytokines (TNF-α, IL-1β and NF-kβ) together with miR-146a-5p downregulation within DPN group.

In the same manner, expression of miR-146a is negatively correlated with caspase-3 expression. Our results showed an increase in caspase-3 level together with decreased expression of miR-146a expression in DPN group. In db/db mice, it is found that downregulation of miR-146a expression led to an increase in IRAK1 and TRAF6 levels, as well as caspase-3, resulting in dorsal root ganglion neuronal death [60]. This demonstrated link among miR-146a-5p, inflammatory and apoptotic markers may elucidate some ambiguity beyond the pathogenesis of DPN.

miR-93 is located at intron 13 of the host minichromosome maintenance 7 gene on chromosome 7q22 [65]. miR-93 is a key regulator of neuronal development in the nervous system, controlling neurite outgrowth and neural stem cell differentiation [66,67]. Also, miR-93 has been reported as an important regulator for inflammation [68]. Another study revealed that miR-93 was involved in regulating inflammatory cytokine expression and neuropathic pain development. Our results came confirmatory showing that miR-93 was significantly downregulated in DPN group compared to T2DM group. Worthley mentioned that all inflammatory cytokines were found to be significantly elevated in our DPN group. This manifests the regulatory correlation of miR-93 on the investigated inflammatory markers and confirms their potential role in the pathogenesis of diabetic neuropathy. A recent report also demonstrated the same regulatory relationship [1].

In the CF group, miR-146a and mir 93 were found to be upregulated compared to both the T2DM and DPN groups. As mentioned above, CF represents a progressive neuropathic condition, and both CF and diabetic neuropathy are closely linked to inflammatory processes. Interestingly, in the CF group, we observed a decrease in inflammatory markers such as NF-kβ, TNF-α, and IL-1β, which may reflect a regulatory correlation with the increased expression of miR-146a and miR-93. However, currently, there are no supportive data in the literature to confirm these findings, likely because CF is rare among diabetic patients. Therefore, further studies with larger sample sizes are needed to validate and expand upon our results.

Correlation analyses provide additional insight into the coordinated molecular alterations associated with progression from uncomplicated T2DM to DPN and ultimately CF. Rather than indicating causal relationships, these correlations reflect stage-specific patterns of interaction between inflammatory pathways, metabolic disturbances, and post-transcriptional regulatory effects of miRNAs.

In patients with T2DM and DPN, multiple correlations were observed between inflammatory markers (including IL-1β, TNF-α, NF-κB, and NLRP3) and metabolic indices such as HbA1c, lipid parameters, and BMI. These findings are consistent with the concept that early and intermediate stages of diabetic neuropathic complications, particularly DPN, are characterized by a close association between metabolic dysregulation and chronic low-grade inflammatory activation, which may contribute to neuropathic progression [2].

Notably, in CF patients, correlation patterns became more selective, with fewer but stronger associations among inflammatory markers, such as the positive correlation between caspase-3 and NF-κB and between NF-κB and Serpin E2. A negative correlation is also seen between IL-1β and NLRP3. This selective pattern may reflect a transition toward tissue-remodeling processes characteristic of advanced neuropathic osteoarthropathy, rather than broad metabolic–inflammatory interactions observed in earlier stages of diabetic complications [3].

A key observation of this study is the consistently strong positive correlations among the investigated miRNAs across all diabetic cohorts, with the highest correlation coefficients observed in CF patients. This coordinated miRNA expression suggests that circulating miRNAs may function as integrated molecular signatures rather than in isolated pathway activation [3,69]. Additionally, this behavior supports their potential role as components of combined biomarker panels rather than as independent diagnostic markers. This guided us toward the evaluation of their combined diagnostic accuracy in the present study.

Clinically, changes seen in the relationships between inflammatory markers and miRNAs across disease stages suggest that the use of combined biomarker profiles may better reflect real disease-stage status. This may aid in raising early clinical suspicion of CF in high-risk diabetic patients, especially when clinical examination and conventional imaging are unavailable.

In the present study, ROC curve analyses were employed to evaluate the discriminative performance of circulating inflammatory biomarkers and miRNAs across different stages of diabetic neuropathic complications, with particular emphasis on distinguishing DPN from CF. The findings demonstrate that both inflammatory markers and circulating miRNAs possess clinically relevant discriminative ability; however, their performance differed depending on the disease stage being evaluated.

For differentiation between T2DM patients without neuropathy and those with DPN, NLRP3 and Serpin E2 exhibited excellent diagnostic accuracy, as reflected by high AUC values. This observation is consistent with the established role of inflammasome activation and inflammatory dysregulation in the early pathogenesis of DN [50]. In contrast, circulating miRNAs demonstrated only modest to moderate discriminative ability in this comparison, suggesting that inflammatory markers may be more sensitive indicators of early neuropathic changes, whereas miRNA dysregulation becomes more pronounced with advancing disease severity.

Notably, the discriminative pattern shifted when comparing DPN patients with CF patients. In this setting, all investigated circulating miRNAs showed good to excellent diagnostic accuracy, with miR-19b-3p demonstrating outstanding performance. This finding suggests that miRNA expression profiles may more accurately reflect the complex molecular alterations associated with advanced neuropathic complications, including dysregulated bone remodeling, and neuroinflammation characteristic of CF [3]. The superior performance of miRNAs in this comparison highlights their potential utility in identifying patients with DPN who may be at increased risk of progressing toward CF.

Importantly, combined biomarker analyses using binary logistic regression further improved discriminative performance for distinguishing CF from DPN compared with individual biomarkers alone. Both the combined inflammatory marker panel (NLRP3 and Serpin E2) and selected miRNA combinations yielded higher AUC values with improved sensitivity and specificity. These findings indicate that integrating multiple biologically related biomarkers may improve the discrimination of CF from DPN, supporting the concept that CF is a multifactorial condition that cannot be adequately characterized by a single molecular marker [3].

Clinically, the observed improvement in discriminative accuracy using combined biomarker panels is particularly relevant, given the well-recognized challenges in the early identification of CF using conventional clinical and radiological approaches [3,5]. While imaging modalities such as MRI remain essential for definitive diagnosis [4,5], circulating biomarker panels may serve as minimally invasive adjunctive tools to support clinical decision-making, especially in patients with equivocal presentations or limited access to advanced imaging. Nevertheless, given the cross-sectional nature of the present study, these findings should be interpreted as evidence of discriminative ability rather than predictive capacity, and longitudinal studies are required to determine whether these biomarkers can prospectively identify patients at highest risk for CF development.

From a clinical perspective, this proposed combined biomarker approach may offer a practical framework for supporting decision-making in patients with DPN and suspected CF. In this context, circulating levels of selected inflammatory markers and miRNAs could be measured from peripheral blood samples, and their values integrated into a combined diagnostic score using the proposed model. Comparison of this score with the identified cutoff value may assist clinicians in identifying patients with DPN who warrant further evaluation for possible CF, particularly in cases with equivocal clinical presentation.

Collectively, all these ROC-based findings support the potential role of circulating miRNA and inflammatory biomarker panels as adjunctive tools for improving the discrimination of advanced neuropathic complications in patients with long-standing diabetes.

### Limitations

This study has several limitations. First, the analysis was limited to circulating plasma biomarkers without tissue-level validation, such as bone biopsy or immunofluorescence studies, which may provide more direct evidence of local inflammatory activity in Charcot neuro-osteoarthropathy. Second, advanced imaging parameters, particularly MRI-based inflammatory assessment (e.g., bone marrow edema or soft tissue enhancement), were not uniformly available for all participants due to limited resources and therefore could not be incorporated into the correlation analysis. Consequently, the identified biomarker alterations should be interpreted as associations with disease status rather than definitive indicators of Charcot-specific pathology. Future studies integrating circulating biomarkers with imaging findings and histopathological validation may further clarify the mechanistic role of these markers in the pathophysiology and activity of CF. Third, the classification of CF relied primarily on clinical diagnosis, rather than the MRI-based staging; therefore, differentiation between acute and chronic phases of Charcot neuroarthropathy was not feasible. Since inflammatory cytokine levels may vary across disease stages, future longitudinal studies incorporating imaging-based staging are needed to better define biomarker dynamics during disease progression. Fourth, the potential influence of comorbid conditions, concomitant medications (e.g., antihypertensive or lipid-lowering agents), metabolic factors such as obesity or blood pressure, and differences in diabetes duration between uncomplicated and complicated diabetic groups cannot be completely excluded and should be considered when interpreting the biomarker associations observed in this study. Fifth, although U6 small nuclear RNA was used for normalization of circulating miRNA expression in this study, which has been reported in previous plasma-based miRNA analyses, future studies should validate these findings using exogenous spike-in controls or multiple stable endogenous reference miRNAs to further improve analytical robustness.

## 5. Conclusions

In conclusion, the present study demonstrates that distinct circulating microRNA expression patterns, together with selected cytokine biomarkers, are associated with progressive stages of diabetic complications, ranging from uncomplicated T2DM to DPN and CF. The combined assessment of circulating miRNAs—particularly miR-19b-3p, miR-451-a, miR-199a-3p, miR-146a-5p, and miR-93-5p—alongside inflammatory cytokines such as NLRP3 and Serpin E2, showed strong discriminative ability in differentiating DPN from uncomplicated T2DM and, more importantly, in distinguishing CF from DPN. These findings suggest that circulating miRNA–inflammatory cytokine signatures may serve as minimally invasive biomarkers that assist in the discrimination of advanced neuropathic complications, particularly CF, among patients with T2DM. Such biomarker profiles may complement current clinical assessment and help support diagnostic evaluation when conventional clinical findings are inconclusive. Although further prospective longitudinal and multicenter studies are required to validate these observations and determine their predictive value, the current results provide evidence supporting the potential clinical relevance of circulating miRNA and inflammatory biomarker signatures as adjunctive tools for characterizing disease-stage and severity in diabetic neuropathic complications.

## Figures and Tables

**Table 1:** Demographic characteristics of the studied cohorts.

Parameters	Control Group (No. = 43)	T2DM Group (No. = 50)	DPN Group (No. = 50)	CF Group (No. = 30)
**Age (year)**	50.70 ± 1	52.72 ± 0.69	53.66 ± 0.73	53.53 ± 1.21
**Disease Duration (years)**	0 ± 0	**3.60 ^A^ ± 0.26**	**16.62 ^AB^ ± 0.84**	**15.17 ^AB^ ± 0.98**
**BMI (Kg/m^2^)**	28.61 ± 0.83	**32.20 ^A^ ± 0.96**	**32.53 ^A^ ± 0.80**	**33.11 ^A^ ± 1.09**
**SEX (M/F)**	17/26	23/27 **^NS^**	25/25 **^NS^**	16/14 **^NS^**
**SBP (mmHg)**	115.81 ± 0.95	119.60 ± 1.24	**137.20 ^AB^ ± 1.03**	**128.00 ^ABC^ ± 2.11**
**DBP (mmHg)**	76.16 ± 0.74	76.80 ± 1.01	**91.60 ^AB^ ± 1.00**	**83.27 ^ABC^ ± 1.61**

All data are expressed as the M ± SEM and approximated to the second digit. Significant data are shown in **bold**. **^A, B, C^**: *p* < 0.05 compared to the control, T2DM, DPN groups respectively using one-way ANOVA followed by Tukey multiple comparison test. **^NS^**: *p* > 0.05 using Chi-square test. BMI: body mass index, BP: blood pressure either systolic (SBP) or diastolic (DBP). Control: healthy control group. T2DM: type 2 diabetes mellitus patients without complications. DPN: diabetic peripheral neuropathy. CF: Charcot foot. M/F: male/female.

**Table 2:** Biochemical characteristics among the control group and different diabetic cohorts.

Parameters	Control Group (No. = 43)	T2DM Group (No. = 50)	DPN Group (No. = 50)	CF Group (No. = 30)
**FPG (mg/dL)**	92.16 ± 1.39	**168.26 ^A^ ± 8.62**	**258.52 ^AB^ ± 9.76**	**280.77 ^AB^ ± 20.14**
**HbA1c (%)**	5.34 ± 0.05	**7.00 ^A^ ± 0.13**	**8.90 ^AB^ ± 0.20**	**9.89 ^ABC^ ± 0.37**
**Cholesterol (mg/dL)**	193.95 ± 5.08	207.18 ± 5.92	211.46 ± 6.55	**163.60 ^ABC^ ± 6.90**
**TGs (mg/dL)**	146.70 ± 12.70	164.64 ± 7.85	**182.00 ^B^ ± 6.15**	**131.07 ^C^ ± 11.39**
**HDL-c (mg/dL)**	47.44 ± 1.17	48.98 ± 1.39	**39.70 ^AB^ ± 0.94**	**45.20 ^C^ ± 2.09**
**LDL-c (mg/dL)**	126.00 ± 3.89	129.26 ± 4.37	135.20 ± 6.21	**94.53 ^ABC^ ± 4.80**
**SCV (m/s)**	NA	37.18 ± 0.48	**31.28** * **± 0.60**	NA
**MCV (m/s)**	NA	61.06 ± 0.90	**34.12** * **± 0.56**	NA

All data are expressed as the M ± SEM and approximated to the second digit. Significant data are shown in **bold**. **^A, B, C^**: *p* < 0.05 compared to the control, T2DM, DPN groups respectively using one-way ANOVA followed by Tukey multiple comparison test. *: *p* < 0.05 using independent Student’s *t*-test. FPG: fasting plasma glucose; HbA1c: glycated hemoglobin; TGs: triglycerides; HDL-c: high-density lipoprotein cholesterol; LDL-c: low-density lipoprotein cholesterol; SCV: sensory nerve conduction velocity; MCV: motor nerve conduction velocity. Control: healthy control group. T2DM: type 2 diabetes mellitus patients without complications. DPN: diabetic peripheral neuropathy. CF: Charcot foot. NA: Not applicable.

**Table 3:** Cytokine biomarkers and miRNA profiles among the control group and different diabetic cohorts.

Level	Parameters	Control Group (No. = 43)	T2DM Group (No. = 50)	DPN Group (No. = 50)	CF Group (No. = 30)
**Serum concentration cytokine levels**	**CASP-3 (pg/mL)**	694.25 ± 9.75	804.74 ± 32.14	**2884.94 ^AB^ ± 168.57**	**1154.04 ^ABC^ ± 80.89**
	**TNF-α (pg/mL)**	158.92 ± 7.20	182.31 ± 5.98	**398.35 ^AB^ ± 29.45**	**261.50 ^AB^ ± 13.42**
	**IL-1β (pg/mL)**	78.76 ± 1.40	83.72 ± 2.07	**178.72 ^AB^ ± 11.79**	**103.99 ^ABC^ ± 4.65**
	**NF-kβ (pg/mL)**	1532.40 ± 43.41	1974.15 ± 101.91	**5327.47 ^AB^ ± 213.49**	**2560.85 ^AC^ ± 207.11**
	**NLRP3 (pg/mL)**	1464.72 ± 56.13	**1951.11 ^A^ ± 87.37**	**4318.18 ^AB^ ± 272.15**	**2488.20 ^AC^ ± 223.97**
	**Serpin E2 (pg/mL)**	1852.02 ± 73.59	1703.21 ± 63.31	**1071.92 ^AB^ ± 26.58**	**1420.06 ^ABC^ ± 84.90**
**Fold expression miRNA levels**	**miR-19b-3p**	1.33 ± 0.24	**0.23 ^A^ ± 0.03**	**0.12 ^A^ ± 0.02**	**0.91 ^BC^ ± 0.16**
	**miR-451-a**	1.32 ± 0.26	**0.24 ^A^ ± 0.04**	**0.09 ^AB^ ± 0.02**	**0.40 ^AC^ ± 0.10**
	**miR-199a-3p**	1.75 ± 0.28	**0.37 ^A^ ± 0.06**	**0.13 ^A^ ± 0.02**	**1.57 ^BC^ ± 0.32**
	**miR-146a-5p**	1.54 ± 0.22	**0.26 ^A^ ± 0.04**	**0.15 ^A^ ± 0.02**	**1.65 ^BC^ ± 0.46**
	**miR-93-5p**	1.88 ± 0.37	**0.24 ^A^ ± 0.04**	**0.09 ^AB^ ± 0.02**	**0.99 ^C^ ± 0.25**

All data are expressed as the M ± SEM of corresponding level and approximated to the second digit. Significant data are shown in **bold**. **^A, B, C^**: *p* < 0.05 compared to the control, T2DM, DPN groups respectively using one-way ANOVA followed by Tukey multiple comparison test. CASP-3: caspase-3; TNF-α: Tumor necrosis factor-α; IL-1β: Interleukin 1β; NF-kβ: Nuclear factor Kabba β; NLRP3: NOD-like receptor pyrin domain 3. Control: healthy control group. T2DM: type 2 diabetes mellitus patients without complications. DPN: diabetic peripheral neuropathy. CF: Charcot foot.

**Table 4:** Parametric linear correlations in-between the investigated parameters among different diabetic cohorts.

Comparison			T2DM Group (No. = 50)	DPN Group (No. = 50)	CF Group (No. = 30)
			r	r	r
Caspase-3	vs.	FPG	**0.30** *	**−0.31** *	*NS*
		SCV	**−0.28** *	*NS*	*NS*
		IL-1β	**0.33** *	**0.31** *	*NS*
		NLRP3	**0.28** *	*NS*	*NS*
		NF-kβ	**0.39** *	**0.41** **	**0.39** *
TNF-α	vs.	IL-1β	*NS*	**0.53** **	*NS*
		NF-kβ	*NS*	**0.39** **	*NS*
		Serpin E2	*NS*	**−0.34** **	*NS*
IL-1β	vs.	FPG	**0.33** *	*NS*	*NS*
		LDL-c	**−0.32** *	*NS*	*NS*
		NF-kβ	**0.34** *	**0.43** **	*NS*
		NLRP3	*NS*	*NS*	**−0.43** *
NF-kβ	vs.	HbA1c	**0.42** **	*NS*	*NS*
		Cholesterol	**−0.34** *	*NS*	*NS*
		LDL-c	**−0.38** *	*NS*	*NS*
		SCV	**−0.44** **	*NS*	*NS*
		Serpin E2	*NS*	*NS*	**0.43** *
NLRP3	vs.	FPG	**0.38** *	*NS*	*NS*
		BMI	*NS*	**0.35** *	*NS*
		HDL-c	*NS*	**0.3** *	*NS*
		Serpin E2	**−0.35** *	*NS*	*NS*
Serpin E2	vs.	TG	*NS*	**−0.31** *	*NS*
		BMI	*NS*	*NS*	**0.42** *
miR-19b-3p	vs.	HDL-c	**−0.3** *	*NS*	*NS*
		miR-451-a	**0.92** **	**0.85** **	*NS*
		miR-199a-3p	**0.79** **	**0.58** **	**0.75** **
		miR-146a-5p	**0.84** **	**0.62** **	**0.86** **
		miR-93-5p	**0.89** **	**0.85** **	**0.81** **
miR-451-a	vs.	Cholesterol	*NS*	**−0.34** *	*NS*
		LDL-c	*NS*	**−0.36** *	*NS*
		miR-199a-3p	**0.62** **	**0.53** **	*NS*
		miR-146a-5p	**0.71** **	**0.56** **	*NS*
		miR-93-5p	**0.87** **	**0.75** **	*NS*
miR-199a-3p	vs.	LDL-c	*NS*	**−0.3** *	*NS*
		SCV	**−0.33** *	*NS*	*NS*
		miR-146a-5p	**0.92** **	**0.73** **	**0.86** **
		miR-93-5p	**0.74** **	**0.62** **	**0.81** **
miR-146a-5p	vs.	Serpin E2	*NS*	*NS*	**0.45** *
		miR-93-5p	**0.78** **	*NS*	**0.81** **

Significant data are shown in **bold** while insignificant data are denoted “*NS*”. *, **: *p* < 0.05, *p* < 0.01 respectively using Pearson linear correlation analysis. A negative sign indicates inverse linear correlation. (**r**): Pearson rank correlation coefficient assuming Gaussian distributions. Control: healthy control group. T2DM: type 2 diabetes mellitus patients without complications. DPN: diabetic peripheral neuropathy. CF: Charcot foot.

**Table 5:** ROC curve analysis data evaluating the discriminative performance for individual and combined biomarkers: NLRP3, Serpin E2 and the Circulating miRNAs under 2 Clinical comparisons; (**A**) to differentiate CF patients from DPN patients (Figure 3B,D) and (Figure 4A–C). (**B**) to differentiate DPN patients from T2DM patients without DN (Figure 3A,C).

Parameter			No.	AUC ± SEM	95% CI	Optimal Cut-Off	Sn (%)	Sp (%)	*p*-Value
(**A**)									
**1-cytokine inflammatory biomarkers**	NLRP3		80	0.80 ± 0.05	[0.70–0.90]	3004.4 *	76%	73.3%	**0.000**
	Serpin E2		80	0.77 ± 0.05	[0.67–0.87]	1089.2 *	56%	90%	**0.000**
	**Predicted combined panel score**		-	0.90 ± 0.04	[0.83–0.97]	0.49 *	88%	83.3%	**0.000**
**2-plasma miRNA signature**	miR-19b-3p		80	0.94 ± 0.03	[0.89–1.00]	1.11 **	90%	85.7%	**0.000**
	miR-451-a		80	0.82 ± 0.06	[0.71–0.93]	0.59 **	70%	82.9%	**0.000**
	miR-199a-3p		80	0.85 ± 0.06	[0.74–0.97]	0.78 **	75%	82.9%	**0.000**
	miR-146a-5p		80	0.83 ± 0.06	[0.71–0.95]	1.27 **	60%	97.1%	**0.000**
	miR-93-5p		80	0.83 ± 0.06	[0.72–0.95]	1.12 **	60%	94.3%	**0.000**
	**Predicted combined panel score**	miR-451-a and miR-146a-5p	-	0.90 ± 0.05	[0.80–0.99]	0.54 **	76%	95%	**0.000**
		miR-451-a and miR-199a-3p	-	0.91 ± 0.04	[0.83–1.00]	0.49 **	78%	97%	**0.000**
(**B**)									
**1-cytokine markers**	NLRP3		100	0.94 ± 0.02	[0.90–0.98]	2965.20 **^#^**	78%	100%	**0.000**
	Serpin E2		100	0.94 ± 0.02	[0.89–0.98]	1297.00 **^#^**	82%	94%	**0.000**
**2-plasma miRNA signature**	miR-19b-3p		100	0.65 ± 0.06	[0.52–0.78]	0.10 **^##^**	100%	88.6%	**0.028**
	miR-451-a		100	0.72 ± 0.06	[0.60–0.84]	0.04 **^##^**	75.7%	62.9%	**0.002**
	miR-199a-3p		100	0.66 ± 0.06	[0.54–0.79]	0.20 **^##^**	45.9%	85.7%	**0.019**
	miR-146a-5p		100	0.61 ± 0.07	[0.48–0.74]	0.25 **^##^**	29.7%	94.3%	0.108
	miR-93-5p		100	0.72 ± 0.06	[0.60–0.84]	0.06 **^##^**	62.2%	74.3%	**0.002**

Significant *p*-values are shown in **bold** indicating discriminative ability of the corresponding biomarker. AUC: area under the ROC curve; the bigger AUC value, the higher the overall accuracy of the corresponding biomarker with respect to its discriminative ability. Sn: sensitivity, Sp: specificity. *, **: for clinical purposes, positive CF patients will be diagnosed if their circulating plasma level of investigated corresponding parameter is below or equal to, above or equal to the optimal cut-off value respectively (i.e., due to CF-associated decreased cytokine concentration and upregulated miRNA expression respectively). **^#^**, **^##^**: for clinical purposes, positive DPN patients will be diagnosed if their circulating plasma level of investigated corresponding parameter is above or equal to, below or equal to the optimal cut-off value respectively (i.e., due to DPN-associated increased cytokine level and downregulated miRNA expression respectively). Combined discriminative performance was done only to differentiate CF patients from DPN as the early detection of CF is challenging and the patient at this stage lacks motivating symptoms and thus needs more non-invasive screening facilities. This is in contrast to DPN patients who were diagnosed easily based on NCS and associated symptoms.

## Data Availability

The data presented in the study are available on request from the corresponding authors.

## Abbreviations

The following abbreviations are used in this manuscript:

ADA	American Diabetes Association
BMI	Body Mass Index
casp-3	Caspase-3
CF	Charcot Foot
CN	Charcot Neuroarthropathy
DBP	Diastolic Blood Pressure
DM	Diabetes Mellitus
DN	Diabetic Neuropathy
DPN	Diabetic Peripheral Neuropathy
FPG	Fasting Plasma Glucose
HbA1c	Glycated Hemoglobin
HDL-c	High-Density Lipoprotein Cholesterol
IL-1β	Interleukin-1β
IRAK	Interleukin-1 Receptor-Associated Kinase
JAK	Janus Kinase
LDL-c	Low-Density Lipoprotein Cholesterol
MAPK	Mitogen-Activated Protein Kinase
MCV	Motor Nerve Conduction Velocity
miRNAs	microRNAs
MRI	Magnetic Resonance Imaging
NCS	Nerve Conduction Study
NF-kβ	Nuclear Factor kappa-β
NIDE	National Institute for Diabetes and Endocrinology
NLRP3	NOD-Like Receptor Pyrin Domain 3
PI3K	Phosphoinositide 3-kinase
PKB	Protein kinase B
ROC	Receiver Operating Characteristic
SBP	Systolic Blood Pressure
SCV	Sensory Nerve Conduction Velocity
Serpin E2	Serpin Peptidase Inhibitor, Clade E, Member 2
STAT	Signal Transducer and Activator of Transcription
T2DM	Type 2 Diabetes Mellitus
TLR	Toll-Like Receptor
TNF-α	Tumor Necrosis factor-α
TRAF	TNF Receptor-Associated Factor
